# Suppression of Human Platelet Activation via Integrin α_IIb_β_3_ Outside-In Independent Signal and Reduction of the Mortality in Pulmonary Thrombosis by Auraptene

**DOI:** 10.3390/ijms20225585

**Published:** 2019-11-08

**Authors:** Chih-Wei Hsia, Cheng-Lin Tsai, Joen-Rong Sheu, Wan-Jung Lu, Chih-Hsuan Hsia, Marappan Velusamy, Thanasekaran Jayakumar, Jiun-Yi Li

**Affiliations:** 1 Graduate Institute of Medical Sciences, College of Medicine, Taipei Medical University, Taipei 110, Taiwan; d119106003@tmu.edu.tw (C.-W.H.); sheujr@tmu.edu.tw (J.-R.S.); d119102013@tmu.edu.tw (C.-H.H.); jayakumar@tmu.edu.tw (T.J.); 2 Graduate Institute of Metabolism and Obesity Sciences, College of Nutrition, Taipei Medical University, Taipei 110, Taiwan; ma48106001@tmu.edu.tw (C.-L.T.); luwj@tmu.edu.tw (W.-J.L.); 3 Department of Pharmacology, School of Medicine, College of Medicine, Taipei Medical University, Taipei 110, Taiwan; 4 Department of Medical Research, Taipei Medical University Hospital, Taipei 110, Taiwan; 5 Department of Chemistry, North Eastern Hill University, Shillong 793022, India; mvelusamy@gmail.com; 6 Department of Cardiovascular Surgery, Mackay Memorial Hospital, Taipei 104, Taiwan; 7 Department of Medicine, Mackay Medical College, New Taipei City 252, Taiwan

**Keywords:** auraptene, human platelet, arterial thrombosis, ERK1/2/JNK1/2, experimental mice

## Abstract

Auraptene is the most abundant coumarin derivative from plants. The pharmacological value of this compound has been well demonstrated, especially in the prevention of cancer and neurodegenerative diseases. Platelet activation is a major factor contributing to arterial thrombosis. Thus, this study evaluated the influence of auraptene in platelet aggregation and thrombotic formation. Auraptene inhibited platelet aggregation in human platelets stimulated with collagen only. However, auraptene was not effective in inhibiting platelet aggregation stimulated with thrombin, arachidonic acid, and U46619. Auraptene also repressed ATP release, [Ca^2+^]i mobilization, and P-selectin expression. Moreover, it markedly blocked PAC-1 binding to integrin α_IIb_β_3_. However, it had no influence on properties related to integrin α_IIb_β_3_-mediated outside-in signaling, such as the adhesion number, spreading area of platelets, and fibrin clot retraction. Auraptene inhibited the phosphorylation of Lyn-Fyn-Syk, phospholipase Cγ2 (PLCγ2), protein kinase C (PKC), Akt, and mitogen-activated protein kinases (MAPKs; extracellular-signal-regulated kinase (ERK1/2), and c-Jun N-terminal kinase (JNK1/2), but not p38 MAPK). Neither SQ22536, an adenylate cyclase inhibitor, nor ODQ, a guanylate cyclase inhibitor, reversed the auraptene-mediated inhibition of platelet aggregation. Auraptene reduced mortality caused by adenosine diphosphate (ADP)-induced pulmonary thromboembolism. In conclusion, this study provides definite evidence that auraptene signifies a potential therapeutic agent for preventing thromboembolic disorders.

## 1. Introduction

Nutritional substances play central roles in the prevention of various inflammatory ailments, including cardiovascular diseases (CVDs). Epidemiologic studies have conveyed a negative relationship between fruit/vegetable intake and risk of CVDs [1]. Arterial thrombosis can lead to the development of CVDs, such as heart attack and even thrombotic stroke. In the case of injury to vascular subendothelial connective tissues, platelets move, adhere at the site of injury, and subsequently initiate vascular thrombosis. Collagen contained in the basement membrane induces a shape change in the platelets, from discoid to spheroid, with pseudopodic projections of platelets [2], and the combined platelet secretion from granules contain substances, such as adenosine triphosphate (ATP), Ca^2+^, and fibrinogen, to strengthen platelet aggregation. This results in a collision of inactivated platelets with those that have undergone the shape change. Such a collision allows the engagement of platelet receptors, which in turn initiates intraplatelet signaling pathways and subsequently activates platelet integrin α_IIb_β_3,_ finally resulting in platelet aggregation and thrombus formation through the binding of soluble fibrinogen and other integrin α_IIb_β_3_ ligands [2]. In resting platelets, integrin α_IIb_β_3_ is normally in a low activation state, unable to interact with fibrinogen. Platelet stimulation with various agonists can induce a conformational change in integrin α_IIb_β_3_, enabling it to bind to its ligands (i.e., fibrinogen, von Willebrand factor), resulting in platelet aggregation onset. This process is known as inside-out signal transduction [3]. Meanwhile, the binding of fibrinogen to active high-affinity integrin α_IIb_β_3_ becomes progressively irreversible, initiating a series of intracellular signaling events, including intracellular calcium mobilization, tyrosine phosphorylation of numerous proteins, activation of phosphoinositide metabolism, and cytoskeleton reorganization. This process is often referred to as outside-in signaling [3].

**Figure 1:**
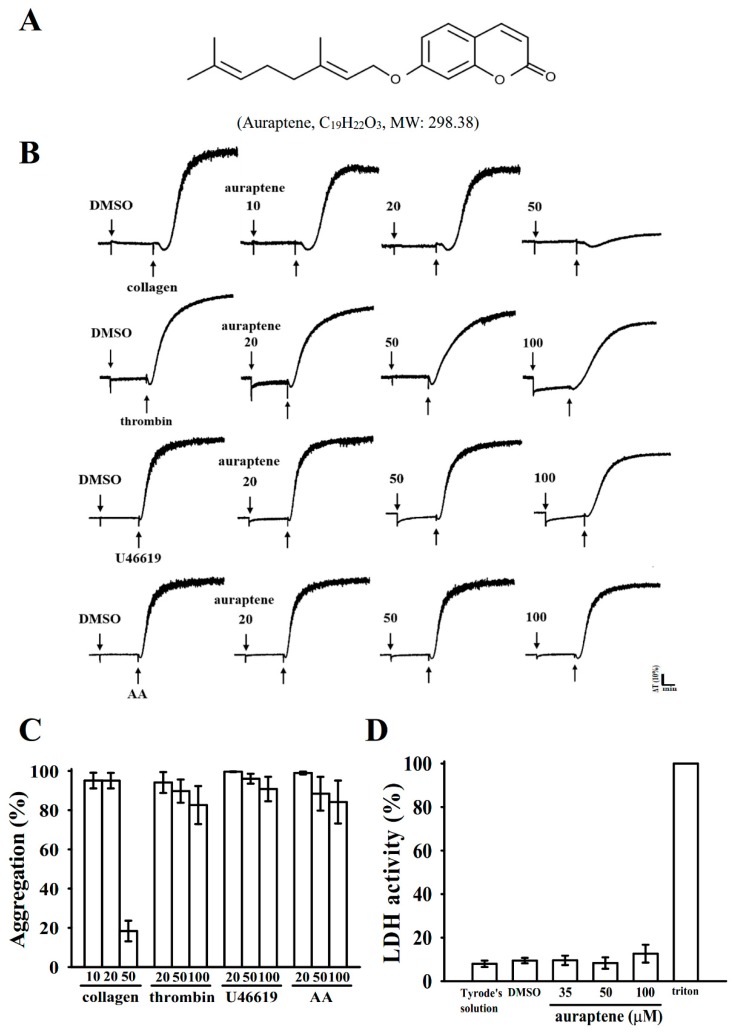
Auraptene inhibits agonists-induced platelet aggregation without causing cytotoxicity. Washed human platelets (3.6 × 10^8^ cells/mL) were preincubated with (**B**) a solvent control (0.1% dimethyl sulfoxide (DMSO)) or auraptene (10–100 μM) (**A**, chemical structure) and subsequently treated with 1 μg/mL collagen, 0.01 U/mL thrombin, 1 μM U46619, or 60 μM arachidonic acid (AA) to trigger platelet aggregation. For the study of cytotoxicity, platelets were preincubated with 0.1% DMSO or auraptene (35, 50, and 100 µM) for 20 min, and a 10-µL aliquot of the supernatant was deposited on the Fuji Dri-Chem slide LDH-PIII. Data (**C**,**D**) are presented as means ± SEM (*n* = 4).

Coumarins, a large class of natural compounds, typically present in the plants of Rutaceae, Apiaceae, and Compositae families. Auraptene (7-geranyloxy coumarin) (Figure 1A) is a well-known coumarin derivative that occurs in several edible fruits and vegetables, especially lemons, grapefruit, and orange [4]. Recently, this compound has attracted considerable research attention because of its potential pharmacological properties. It inhibits inflammatory responses and prevents several types of cancer, neurodegenerative diseases, and cerebral ischemic infarction [5,6]. Studies have also discovered that by increasing nitric oxide bioavailability and alleviating vascular endothelial dysfunction, auraptene has hypotensive effects [7].

A bioassay-guided fraction separation study found that the isolation of seven coumarin compounds, including auraptene, had strong inhibitory activity on rabbit platelet aggregation induced by collagen, arachidonic acid (AA), and platelet-activating factor (PAF) [8]. Auraptene also possesses marked antiplatelet activity in collagen, thrombin, and ADP-induced rabbit platelets with a 50% inhibitory concentration (IC50) of approximately 100 to 200 µM [9]. It has been proposed that coumarin compounds have high lipid solubility and bind to plasma protein [10]. The lipophilic properties of coumarins may enhance their permeability into the cells and stimulate biological activities. Equally, our initial screening study demonstrated that 50 µM coumarin-derived auraptene significantly inhibited aggregation in washed human platelets. This result led us to conduct a thorough investigation on the effect of auraptene on human platelet activation. Specifically, we studied the detailed mechanisms underlying the inhibitory effects of auraptene on platelet activation both ex vivo and in vivo.

## 2. Results

### 2.1. Inhibitory Profiles of Auraptene in Agonist-Stimulated Washed Human Platelets

Auraptene is a coumarin-derived compound from citrus plants, and it possesses a geranyloxyl moiety at the C-7 position (Figure 1A). Teng et al. [9] reported that auraptene (100–200 µM) concentration dependently suppressed collagen, thrombin, ADP, AA, U46619 (a thromboxane A_2_ receptor agonist), and platelet-activating factor-stimulated rabbit platelet aggregation. No further evidence has been provided after that study. In this study, auraptene markedly inhibited collagen (1 µg/mL)-stimulated human platelet aggregation at 10 to 50 µM concentrations. These concentrations are lower than those employed for rabbit platelets in a previous study [9]. However, auraptene slightly inhibited platelet aggregation, and the inhibition was not significant in platelets stimulated with either AA, thrombin, or U46619, even with concentrations up to 100 µM (Figure 1B,C). These results indicate that auraptene exhibited differences on its potency and mechanisms between the human and rabbit platelets. The IC_50_ of auraptene in collagen-induced platelet aggregation was approximated at 35 μM (Figure 1C). The solvent control (0.1% DMSO) did not exhibit any significant effects on platelet aggregation (Figure 1B) In addition, auraptene (50 µM) inhibited ADP (20 µM)-induced platelet aggregation by approximately about 20% in platelet-rich plasma (data not shown). Furthermore, the lactate dehydrogenase (LDH) assay revealed that auraptene (35, 50, and 100 μM) pretreatment for 20 min did not alter LDH release and did not cause any observable cytotoxic effects in platelets (Figure 1D). This result demonstrates that auraptene neither affects platelet permeability nor induces platelet cytolysis.

### 2.2. Regulatory Characteristics of Platelet Activation by Auraptene

**Figure 2:**
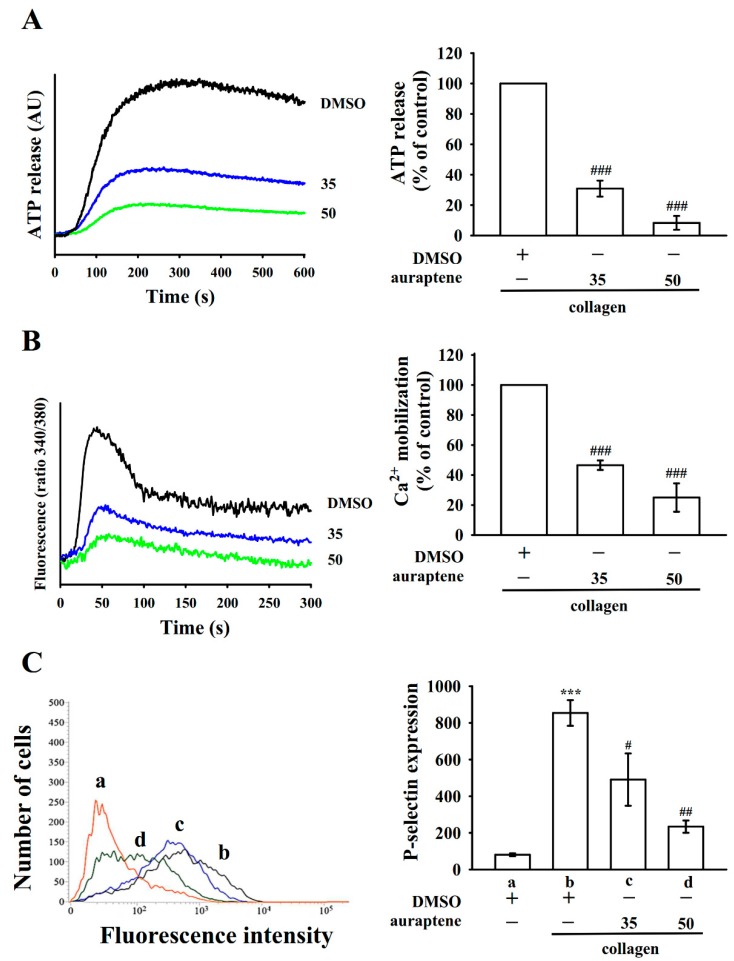
Auraptene reduces collagen-stimulated ATP release, relative [Ca^2+^]i mobilization, and surface P-selectin expression in human platelets. Washed platelets (3.6 × 10^8^ cells/mL) were preincubated with 0.1% DMSO or auraptene (35 and 50 µM), followed by the addition of collagen (1 μg/mL) to trigger (**A**) ATP release (AU; arbitrary unit), (**B**) relative [Ca^2+^]i mobilization, and (**C**) surface P-selectin expression (**a**) Resting platelets (DMSO-treated) or platelets were preincubated with 0.1% DMSO (**b**) or auraptene (**c**, 35; **d**, 50 µM), as defined in the materials and methods. The corresponding statistical data are displayed on the right panel of each figure. Data are presented as mean ± SEM (*n* = 4). *** *p* < 0.001 compared with the resting control; ^#^ *p* < 0.05, ^##^ *p* < 0.01, and ^###^ *p* < 0.001, compared with the 0.1% DMSO-treated group.

Platelet activation is associated with the release of granular contents (e.g., ATP and Ca^2+^ release from dense granules and P-selectin expression from α-granules), thus causing abundant platelet aggregation. As shown in Figure 2A, auraptene (35 and 50 µM) concentration dependently moderated ATP release in collagen (1 μg/mL)-stimulated platelets. In addition, collagen-stimulated [Ca^2+^]i was prevented by 35 and 50 μM auraptene. This was approximated at 50% and 75%, respectively (Figure 2B). P-selectin is placed on the inside wall of α-granules in quiescent platelets, and platelet stimulation releases α-granules, which leaks the inside walls of the granules on the outside of the cells [11]. Here, auraptene prominently diminished collagen-stimulated P-selectin expression (resting, 80.3 ± 7.8; collagen-induced, 854.3 ± 70.1; auraptene at 35 μM, 490.6 ± 142.7 and 50 μM, 234.0 ± 33.5; *n* = 4; Figure 2C).

### 2.3. Role of Auraptene in Integrin α_IIb_β_3_ Activation

**Figure 3:**
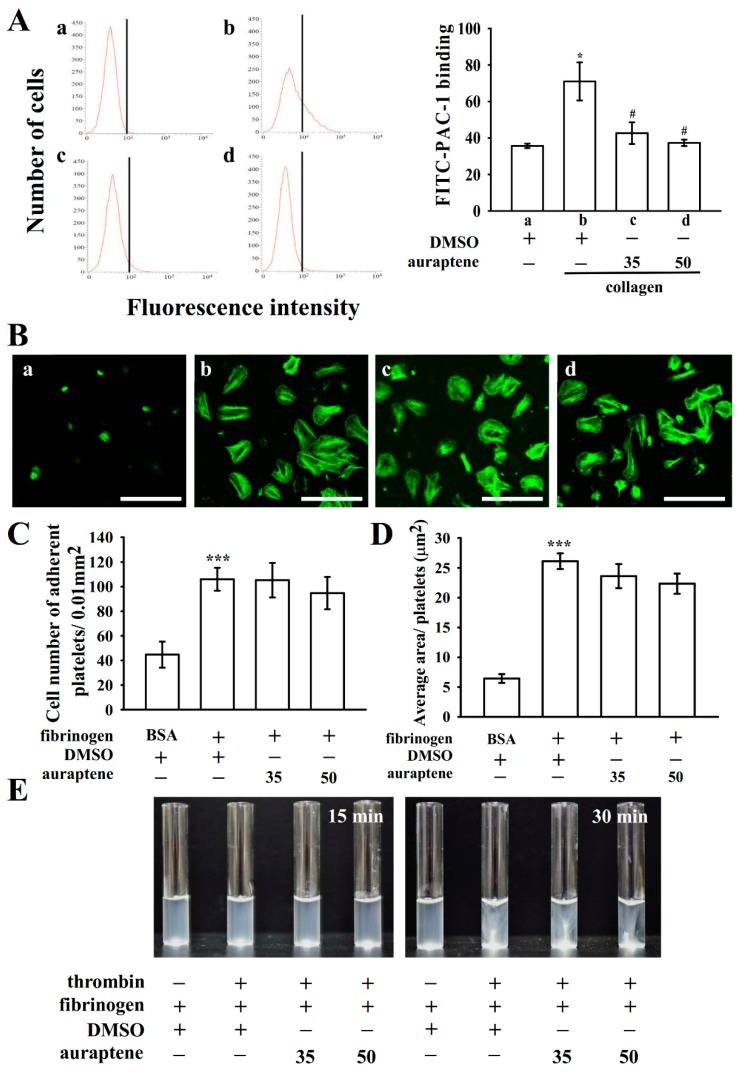
Controlling activities of auraptene on integrin α_IIb_β_3_ activation, platelet adhesion, and spreading on immobilized fibrinogen as well as clot retraction. (**A**) Resting platelets (DMSO-treated) (a) or platelets were preincubated with 0.1% DMSO (b) or auraptene (c, 35; d, 50 µM) and the FITC-conjugated anti-PAC-1 mAb (2 µg/mL) were added before the addition of collagen for flow cytometer analysis. For other experiments, (**B**) washed platelets were allowed to spread on the (a) Bovine serum albumin (BSA)- or (b–d) fibrinogen-coated surfaces with (b) solvent control (0.1% DMSO) or auraptene (c, 35; d, 50 μM). Platelets were consequently labeled with FITC-conjugated phalloidin, as designated in the materials and methods. (**C**) The number of adherent platelets per 0.01 mm^2^ and (**D**) the average spreading surface area of individual platelets in sight views are plotted. (**E**) Washed platelets were suspended in 2 mg/mL fibrinogen with 0.1% DMSO or auraptene (35 and 50 μM) before the thrombin (0.01 U/mL) stimulation. Images were photographed at 15- and 30-min intervals. Profiles in (**E**) are representative of four similar experiments. Data are presented as mean ± SEM (*n* = 4). (**A**) * *p <* 0.05 and ^#^ *p <* 0.05, compared with the resting and DMSO-treated groups, respectively; (**C**,**D**) *** *p <* 0.001, compared with the immobilized BSA group.

Platelet aggregation is dependent on fibrinogen–integrin α_IIb_β_3_ interaction. Integrin α_IIb_β_3_ inhibition results in the disaggregation of accumulated platelets [12]. To know whether auraptene disturbs integrin α_IIb_β_3_ activation, the binding of the fluorescein isothiocyanate (FITC)-conjugated PAC-1 mAb that responds to the stimulation-induced conformational epitope of integrin α_IIb_β_3_ was analyzed by flow cytometry (Figure 3A). Auraptene (35 and 50 µM) considerably inhibited integrin α_IIb_β_3_ activation stimulated by collagen. This finding indicates that auraptene may influence the binding of PAC-1 to the activated integrin α_IIb_β_3_. Furthermore, the quantity of immobilized fibrinogen to which platelets adhered was significantly greater than that of immobilized bovine serum albumin (BSA) (Figure 3Ba,b), as indicated by the presence of platelets stained with FITC-conjugated phalloidin. No significant differences were found in platelet adhesion and spreading on immobilized fibrinogen between 0.1% DMSO and auraptene (35 and 50 µM)-treated platelets (Figure 3Bc,d). Figure 3C shows resting platelets were fixed to immobilized fibrinogen (106.0 ± 9.3 platelets/0.01 mm^2^; *n* = 4) compared to the immobilized BSA (BSA, 44.8 ± 10.6 platelets/0.01 mm^2^; *n* = 4). However, platelets that had been treated with auraptene showed similar binding effects to the fibrinogen-coated surface (35 µM, 105.3 ± 14.3 platelets/0.01 mm^2^; 50 µM, 94.8 ± 13.1 platelets/0.01 mm^2^, *n* = 4). Moreover, no significant difference was found in the surface coverage of a single platelet between 0.1% DMSO and auraptene-treated platelets (0.1% DMSO, 26.1 ± 1.3 µm^2^; auraptene 35 µM, 23.6 ± 2.0 µm^2^ and 50 µM, 22.4 ± 1.7 µm^2^; *n* = 4). Furthermore, clot retraction of fibrin polymers, the end process in thrombus formation, is necessary in aggregate stabilization [3], and it is paradigmatic of integrin α_IIb_β_3_ outside-in signaling. A clot retraction study was performed by adding thrombin to a solution enclosing fibrinogen in the presence of either auraptene- or 0.1% DMSO-treated human platelets. The results show that clot retraction was more apparent after the 30-min incubation of 0.1% DMSO than that of the 15-min incubation in platelets (Figure 3E). However, clot retraction was not significantly abridged in platelets treated in 35 and 50 µM auraptene. This finding indicates that auraptene has no significant ability to reduce fibrin clot retraction. Taken together, the results indicate that auraptene does not restrict clot retraction as well as the integrin α_IIb_β_3_-mediated outside-in signaling of cell adhesion and spreading.

### 2.4. Effects of Auraptene on Fyn, Lyn, Syk, and PLCγ2/PKC Signaling

**Figure 4:**
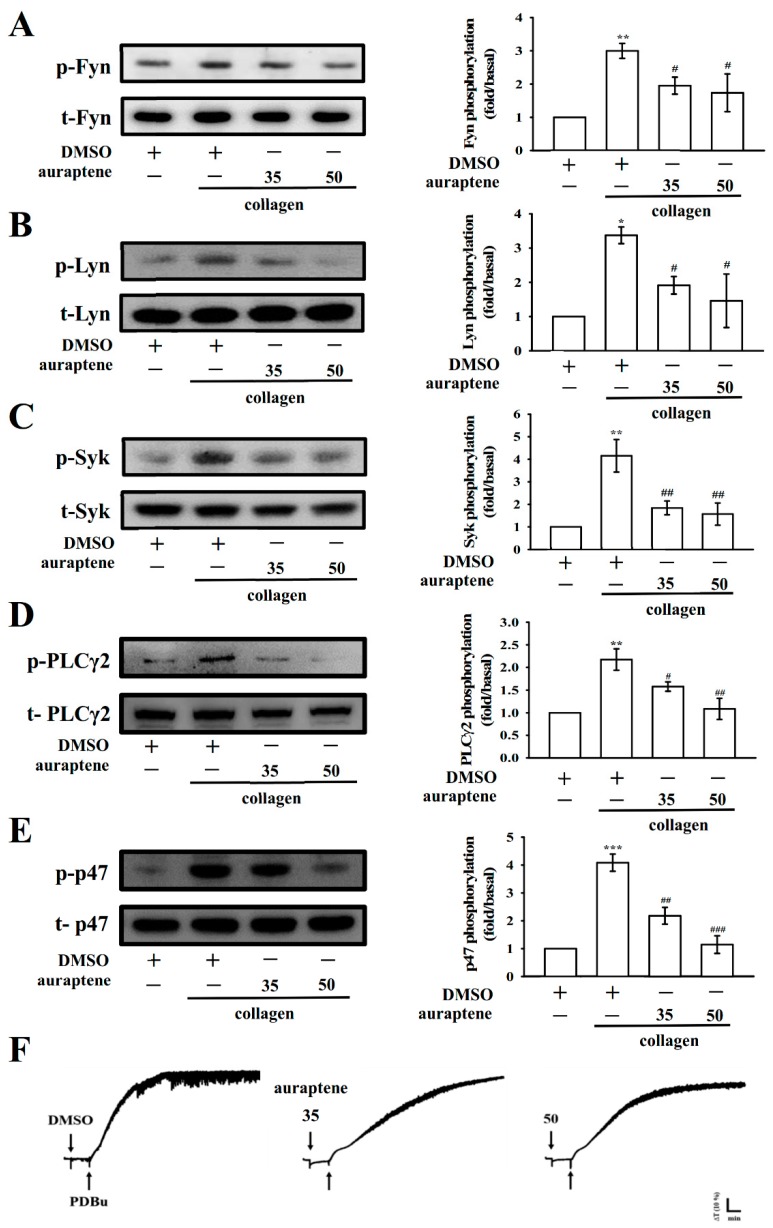
Regulatory effects of auraptene on Fyn, Lyn, Syk, and PLCγ2/PKC phosphorylation in platelets. Washed platelets were preincubated with 0.1% DMSO or auraptene (35 and 50 μM) and then treated with collagen (1 µg/mL) or PDBu (150 nM) to trigger either (**A**) Fyn, (**B**) Lyn, (**C**) Syk, (**D**) PLCγ2, (**E**) PKC activation (p-p47), or (**F**) platelet aggregation. Data are given as mean ± SEM (*n =* 4). * *p* < 0.05, ** *p* < 0.01, and *** *p* < 0.001, compared with the resting platelets; ^#^ *p* < 0.05, ^##^ *p* < 0.01, and ^###^ *p* < 0.001, compared with the DMSO-treated group. Profiles in (**F**) are illustrative of three independent experiments.

The binding of collagen with glycoprotein (GP) VI is reported to be mediated by the Src-family kinases (SFKs) Fyn and Lyn [13], resulting in the activation of the cytosolic tyrosine kinase Syk. As shown in Figure 4A–C, the collagen-induced phosphorylation of Fyn, Lyn, and Syk was significantly inhibited by auraptene. Moreover, PLC comprises a family of kinases that hydrolyze phosphatidylinositol 4,5-bisphosphate [PI(4,5)P2] to produce two second messengers, diacylglycerol (DAG) and inositol trisphosphate (IP_3_). DAG activates PKC-stimulating protein phosphorylation (p47 protein; pleckstrin) and ATP release in activated platelets; IP_3_ elevates calcium influx [14]. In the present study, the effect of auraptene on the phosphorylation of the PLCγ2-PKC signaling cascade was examined. Auraptene treatment (35 and 50 µM) concentration dependently diminished the phosphorylation of PLCγ2 in collagen-activated platelets (Figure 4D). As illustrated in Figure 4E, a similar molecular weight protein p47 (47 kDa) was apparently phosphorylated in collagen-activated platelets, which was concentration-dependently reduced by auraptene. In addition, neither 35 nor 50 µM of auraptene reduced PDBu (PKC activator)-induced platelet aggregation (Figure 4F). This result indicates that auraptene inhibits both SFKs and PLCγ2/PKC activation.

### 2.5. Auraptene Inhibits Akt, ERK1/2, and JNK1/2 Activation

**Figure 5:**
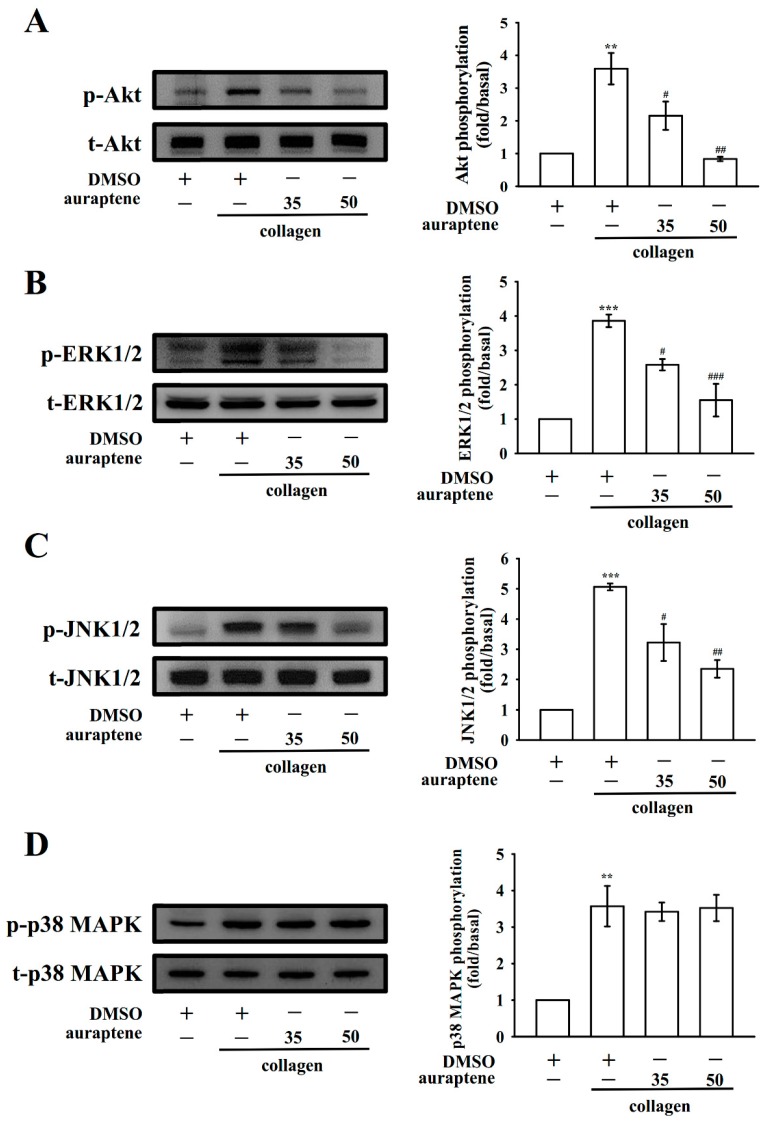
Effects of auraptene on Akt, ERK1/2, JNK1/2, and p38 MAPK phosphorylation in collagen-activated platelets. Washed platelets were preincubated with 0.1% DMSO or auraptene (35 and 50 μM) and then treated with collagen (1 µg/mL) for (**A**) Akt, (**B**) ERK1/2, (**C**) JNK1/2, and (**D**) p38 MAPK phosphorylation. Data are given as mean ± SEM (*n* = 4). ** *p* < 0.01 and *** *p* < 0.001, compared with the resting platelets; ^#^ *p <* 0.05, ^##^ *p* < 0.01, and ^###^ *p* < 0.001, compared with the DMSO-treated group.

The Akt (protein kinase B; Ser-Thr kinase) pathway, also termed the phosphatidylinositol 3-kinase (PI3K)-Akt pathway, mediates downstream responses, such as cell survival, growth, proliferation, and platelet activation [15]. In this study, at concentrations of 35 and 50 μM, auraptene markedly inhibited the phosphorylation of Akt stimulated by collagen (Figure 5A). The antiplatelet mechanisms of auraptene were examined by investigating several MAPK molecules, such as ERK1/2, JNK1/2, and p38 MAPK. These molecules regulate major cellular functions, such as cell proliferation, gene expression, cell differentiation, cell survival, and platelet activation [16]. In this study, auraptene inhibited the phosphorylation of ERK1/2 (Figure 5B) and JNK1/2 (Figure 5C) but not the phosphorylation of p38 MAPK (Figure 5D). This result indicates that inhibition of the Akt, ERK1/2, and JNK1/2 signaling pathways may play a crucial role in auraptene-mediated antiplatelet effects.

### 2.6. Effectiveness of Auraptene in Cyclic Nucleotide Formation and Acute Pulmonary Thromboembolism In Vivo

**Figure 6:**
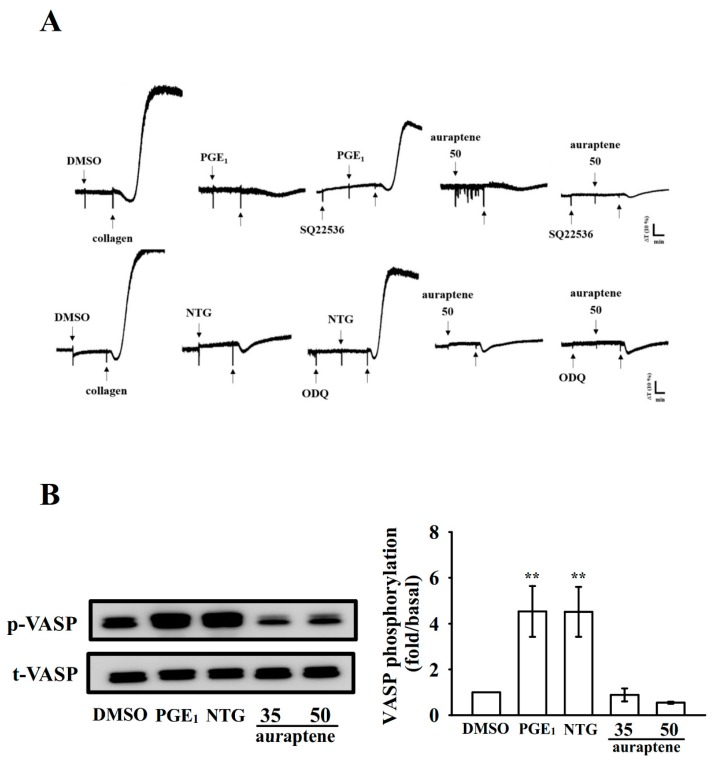
Effectiveness of auraptene in cyclic nucleotides and vasodilator-stimulated phosphoprotein (VASP) phosphorylation as well as acute pulmonary thrombosis. Washed platelets were preincubated with (**A**) prostaglandin E_1_ (PGE_1_; 10 µM), nitroglycerin (NTG; 10 µM), or auraptene (50 μM) in the presence of SQ22536 (100 μM) or ODQ (10 µM) 3 min before the addition of collagen (1 μg/mL) to activate platelet aggregation. (**B**) Washed platelets were preincubated with 0.1% DMSO, PGE_1_ (1 μM), NTG (10 μM), and auraptene (35 and 50 μM) for immunoblotting the Vasodilator-stimulated phosphoprotein (VASP) phosphorylation. Profiles in (A) are representative of four independent experiments. Data in (**B**) are presented as mean ± SEM (*n* = 4). ** *p* < 0.01, compared with the 0.1% DMSO-treated group.

Cyclic AMP and cyclic GMP are critical second messengers that regulate multiple targets, such as cyclic AMP- or cyclic GMP-dependent protein kinases. These kinases are involved in the phosphorylation of vasodilator-stimulated phosphoprotein (VASP). As shown in Figure 6A, both SQ22536 (100 μM), an adenylate cyclase inhibitor, and ODQ (10 μM), a guanylate cyclase inhibitor, significantly reversed the PGE_1_ (1 μM)- and NTG (10 μM)-mediated inhibition of collagen-induced platelet aggregation, respectively. Neither SQ22536 nor ODQ significantly reversed the auraptene (50 μM)-mediated inhibition of platelet aggregation. In addition, both PGE_1_ (1 μM) and NTG (10 μM) obviously trigger VASP phosphorylation, whereas auraptene (35 and 50 μM) had no effect in this reaction (Figure 6B). These results indicate that the antiplatelet mechanisms of auraptene may be independent of cyclic nucleotide formation.

Moreover, the antithrombotic effects of auraptene on acute pulmonary embolism-induced mortality in mice are summarized in Table 1. The results revealed that auraptene at 7.5 and 15 mg/kg significantly reduced ADP-induced mortality and significantly reversed platelet numbers in mice. Specifically, the reduction rate was from 87.5% (7 dead, *n* = 8 in total) in 0.1% DMSO-treated mice to 62.5% (5 dead, *n* = 8 in total) and 25% (2 dead, *n* = 8 in total) in auraptene-treated mice, respectively. Furthermore, aspirin (20 mg/kg), an effective antiplatelet drug prescribed for preventing or treating cardiovascular diseases, also reduced mortality and reversed platelet numbers in this study (Table 1).

## 3. Discussion

This study is the first to demonstrate that in addition to its well-known properties, auraptene possesses marked antiplatelet activity against human platelets. Because it is a hydrophobic molecule, auraptene is sparingly soluble in aqueous buffers. Thus, when administered orally, it undergoes rapid absorption and is rapidly distributed to all tissues; it is especially able to penetrate the blood–brain barrier [7]. Furthermore, auraptene is widely and easily available in large amounts through chemical synthesis. Therefore, auraptene is a potentially new therapeutic agent for the treatment of thrombotic diseases in humans.

In this study, auraptene potently inhibited collagen-induced platelet aggregation. However, inhibition was slight and not statistically significant for platelets stimulated with other agonists. This finding implies that auraptene-mediated inhibition of platelet aggregation occurs through a distinct PLC-dependent mechanism. The collagen-induced platelet activation noticeably modifies phospholipase activation. The triggering of PLC causes IP_3_ and DAG formation, which in turn activates PKC and subsequently induces the phosphorylation of the p47 protein [17]. The IP_3_-triggering calcium release from the intracellular calcium store is only a minor part; however, the predominant part is regulated by the calcium channels on the plasma membrane of platelets [18]. Therefore, auraptene seems not only to affect IP_3_-mediated calcium exflux but also affects calcium influx from calcium channels. The PLC enzyme comprises six families: PLCβ, PLCγ, PLCδ, PLCε, PLCζ, and PLCη [19]. The PLCγ family comprises isozymes 1 and 2. PLCγ2 participates in collagen-dependent signaling in platelets [19]. In our current study, auraptene weakened collagen-activated PLCγ2. However, auraptene exerted no direct effects on PKC activation because it did not reduce PDBu-induced platelet aggregation. This finding suggests that PLCγ2 downstream signaling involves auraptene-mediated inhibition of platelet activation.

GP VI belongs to a membrane of the immunoglobulin superfamily, which forms a complex with the Fc receptor γ-chain (FcRγ) containing immunoreceptor tyrosine-based activation motifs (ITAM) and is phosphorylated by SFKs, such as Fyn and Lyn [2]. In platelets, SFKs, particularly Lyn and Fyn, play a crucial role downstream of collagen receptors [13]. Studies have also proven that SFKs play a role in thromboxane generation, shape change, as well as the regulation of phosphorylation of Akt [20] and ERK [21]. In addition, MAPKs constitute a family of serine-threonine protein kinases that convert extracellular stimuli into a wide range of cellular responses. Previous studies using specific inhibitors or knockout mice have identified ERK1/2, JNK1/2, and p38 MAPK in platelets and showed their participation in platelet activation [22]. All these MAPKs were triggered by specific MAPK kinases (MEKs). The physiopathological aspects of JNK1/2 and ERK1/2 in platelets remain largely unknown. However, proof advocates that the destruction of α_IIb_β_3_ integrin activation may be intricate in their roles [23]. Moreover, ERK1/2 activation is essential for collagen-induced platelet aggregation [24]. Cytosolic phospholipase A_2_ (cPLA_2_), which catalyzes AA release to produce thromboxane A_2_, is an important substrate of p38 MAPK activation and is tempted by different stimulators, including thrombin [25]. The present results indicate that auraptene-promoted inhibition of collagen-stimulated platelet activation involves ERK1/2 and JNK1/2 but not p38 MAPK. This might explain why auraptene displays potency in inhibiting platelets stimulated with collagen compared to AA, U46619, and thrombin. Moreover, Fan et al. [22] recently reported that ERK1/2 and JNK1/2, but not p38 MAPK, are the major MEK kinase (MEKK)3 downstream signaling molecules in platelet activation. Thus, we speculate that auraptene may affect MEEK3, resulting in the inhibition of ERK1/2 and JNK1/2 phosphorylation. However, this needs to be proven in a future study.

Akt is a downstream regulator of PI3K, and Akt-deleted mice showed weaknesses in platelet activation [26]. Hence, Akt activation through protein kinases, particularly PI3K, may be attractive targets for the development of antithrombotic therapeutics. Although the effectors through which Akt contributes to platelet activation are not definitively known, several candidates, including glycogen synthase kinase 3β, phosphodiesterase 3A, and integrin β_3_, have been identified [26]. In addition, both PI3K/Akt and MAPKs are mutually activated in platelets, and PKC’s role as an upstream regulator of them has also been reported [27].

Cyclic AMP and cyclic GMP elevation in platelets activates their respective protein kinase A and protein kinase G. This in turn regulates platelet activation by phosphorylating intracellular protein substrates, such as VASP. Such protein substrates are involved in the inhibition of agonist-induced platelet aggregation and the adhesion of platelets to the vascular wall [28]. Increased levels of cyclic nucleotides hinder several platelet responses and diminish the levels of [Ca^2+^]i through Ca^2+^ uptake into the dense tubular system. This inhibits the stimulation of PLC/PKC signaling. Thus, increased cyclic AMP/GMP mutually work in the inhibition of platelet triggering. In this study, both SQ22536 and ODQ did not significantly reverse the auraptene-mediated reduction of platelet aggregation. Auraptene also had no effect on VASP phosphorylation. Therefore, the intracellular cyclic nucleotides–VASP pathway is not involved for the noted antiplatelet effects of auraptene.

The binding of fibrinogen to integrin α_IIb_β_3_ is a major component of the platelet aggregation response. Integrin α_IIb_β_3_ undergoes conformational changes upon activation. It generates specific ligand-binding sites for fibrinogen, von Willebrand factor, and fibronectin [3]. Platelet adheres to immobilized fibrinogen and mediates clot retraction, which are involved in integrin α_IIb_β_3_ outside-in signaling and cytoskeleton reorganization [3]. Here, auraptene reduced the binding of PAC-1 to activated integrin α_IIb_β_3_. However, it had no effects on clot retraction and abolishing platelet adhesion and spreading. This indicates that auraptene influences integrin α_IIb_β_3_ in inside-out, but not outside-in, signaling. Animal models of vascular thrombosis are necessary to understand the effectiveness of test compounds to prevent or treat these diseases. An ideal mouse model should be technically simple, fast to establish, and easily reproducible. An intravenous injection of platelet agonist ADP results in the formation of acute occlusive thrombi; the mortality rate of ADP-treated mice is believed to result in the formation of thrombi in lungs [29]. In this study, the mortality rate of auraptene-treated animals was markedly lower than that of solvent-treated animals. This indicates that auraptene is a natural compound that potentially treats thromboembolic disorders.

In conclusion, auraptene shows a unique property of inhibiting human platelet activation. Thus, it has potential therapeutic applications. Specifically, auraptene significantly inhibits platelet triggering by delaying the SFKs and PLCγ2-PKC cascade. This can lead to diminished activation of Akt and ERK1/2/JNK1/2. This inhibits the release of substances, such as P-selectin, ATP, and [Ca^2+^]i, which in turn influences integrin α_IIb_β_3_ inside-out signaling and eventually inhibits platelet aggregation. The results of this study provide new insights into the role of auraptene in human platelet activation.

## 4. Materials and Methods

### 4.1. Chemicals and Reagents

Collagen (type I), luciferin-luciferase, arachidonic acid (AA), U46619, ADP, fibrinogen, phorbol-12, 13-dibutyrate (PDBu), SQ22536, ODQ, heparin, prostaglandin E_1_ (PGE_1_), FITC-phalloidin, nitroglycerin (NTG), and thrombin were purchased from Sigma (St. Louis, MO, USA). Auraptene (>98%) was purchased from Cayman Chem. (Ann Arbor, MI, USA). Fura 2-AM was purchased from Molecular Probes (Eugene, OR, USA). The anti-phospho-p38 mitogen-activated protein kinase (MAPK) Ser^182^ monoclonal antibody (mAb) was purchased from Santa Cruz (Santa Cruz, CA, USA). Anti-p38 MAPK, anti-phospho-JNK (Thr^183^/Tyr^185^) and anti-phospho-Syk (Tyr^525^/^526^) polyclonal antibodies (pAbs), anti-phospholipase C (PLC)γ2, anti-phospho(Tyr^759^) PLCγ2, and anti-Syk mAb and anti-phospho-p44/p42 extracellular signal-regulated kinase (ERK) (Thr^202^/Tyr^204^) pAbs were purchased from Cell Signaling (Beverly, MA, USA). Anti-phospho-Akt (Ser^473^) and anti-Akt mAbs were purchased from Biovision (Mountain View, CA, USA). Anti-Lyn mAb, anti-Fyn mAb, and anti-phospho-Fyn (Y^530^) pAbs and anti-phospho-Lyn (Y^507^) mAb were obtained from Abcam (Cambridge, UK). The anti-α-tubulin mAb was purchased from NeoMarkers (Fremont, CA, USA). FITC-anti-human CD42P (P-selectin) and FITC-anti-human CD41/CD61 (PAC-1) mAbs were obtained from BioLegend (San Diego, CA, USA). A Hybond-P polyvinylidene difluoride (PVDF) membrane, the enhanced chemiluminescence Western blotting detection reagent, horseradish peroxidase (HRP)-conjugated donkey anti-rabbit immunoglobulin G (IgG), and sheep anti-mouse IgG were purchased from Amersham (Buckinghamshire, UK). A 0.1% dimethyl sulfoxide (DMSO) solution was used to prepare the auraptene suspension.

### 4.2. Platelet Preparation, Aggregation, and ATP Release

This study conformed to the directives of the Helsinki Declaration and was approved by the Institutional Review Board of Taipei Medical University (TMU-JIRB-N201812024,09 January 2019). An informed consent form was provided to all human blood donors involved this study. Washed human platelets were prepared as described previously [30]. Either auraptene (10–100 μM) or solvent control (0.1% DMSO) was incubated with platelets for 3 min before stimulation. ATP release was assayed using a Hitachi Spectrometer F-7000 (Tokyo, Japan) based on the manufacturer’s procedure.

### 4.3. Intracellular [Ca^2+^] Mobilization Using Fura 2-AM Fluorescence

To measure the level of [Ca^2+^]i, citrated whole blood was centrifuged and the supernatant was incubated with 5 μM Fura 2-AM. The Fura 2-AM fluorescence was measured using the Hitachi Spectrometer F-7000. The intracellular calcium ([Ca^2+^]i) level was calculated at the excitation wavelengths of 340 and 380 nm and the emission wavelength of 500 nm [31].

### 4.4. Lactate Dehydrogenase Assay

The cytotoxic effect was examined by determining the level of lactate dehydrogenase (LDH). Washed platelets (3.6 × 10^8^ cells/mL) were preincubated with either auraptene (35, 50, and 100 µM) or 0.1% DMSO for 20 min at 37 °C. An aliquot of the supernatant (10 µL) was deposited on a Fuji Dri-Chem slide LDH-PIII (Tokyo, Japan) and read by a spectrophotometer (UV-160; Shimazu, Japan). The maximal level of LDH was observed in triton-treated platelets.

### 4.5. Flow Cytometry Analysis for P-Selectin Expression and Integrin α_IIb_β_3_ Activation

Washed platelets (3.6 × 10^8^ cells/mL) were preincubated with a combination of auraptene (35 and 50 µM) and the FITC-conjugated anti-P-selectin mAb (2 µg/mL) or PAC-1 mAb (2 µg/mL) for 3 min. They were then activated by collagen (1 µg/mL). The suspensions were subsequently assayed for fluorescein-labeled platelets on a flow cytometer (FACScan system, Becton Dickinson, San Jose, CA, USA).

### 4.6. Platelet Adhesion and Spreading Analysis on Immobilized Fibrinogen

Analysis of platelet spreading on immobilized fibrinogen was performed by using confocal microscopy as described previously [32]. Platelets were stained with FITC-labeled phalloidin and imaged using a Leica TCS SP5 microscope equipped with a 100×, 1.40 NA oil immersion objective (Leica, Wetzlar, Germany). Platelet adhesion and the platelet spreading were analyzed using NIH ImageJ software (NIH, Bethesda, MD, USA; http://rsbweb.nih.gov/ij/).

### 4.7. Platelet-Mediated Clot Retraction

Washed platelets were remixed in Tyrode’s solution that contained 2 mg/mL fibrinogen and 1 mM CaCl_2_ in tubes designed for aggregation [33]. The platelet suspensions were preincubated with auraptene (35 and 50 µM) or 0.1% DMSO for 3 min prior to thrombin (0.01 U/mL)-induced clot retraction without stirring. The reaction was developed at 37 °C in an aggregometer tube and photographed at 15 and 30 min, respectively.

### 4.8. Immunoblotting

Washed platelets (1.2 × 10^9^ cells/mL) were pretreated with either auraptene (35 and 50 µM) or 0.1% DMSO, and collagen was subsequently added to trigger platelet activation. The platelet suspensions were lysed and separated through 12% SDS-PAGE. Several proteins were detected using specific primary antibodies. The respective semi quantitative results were obtained by quantifying the optical density of protein bands on a video densitometer and through Bio-profil Biolight software, Version V2000.01 (Vilber Lourmat, Marne-la-Vallée, France).

### 4.9. Acute Pulmonary Thromboembolism Stimulated by ADP in Mice

Acute pulmonary thromboembolism was induced in mice following a previous method [34]. Mice were intraperitoneally administered different doses of auraptene (7.5 and 15 mg/kg) and aspirin (20 mg/kg) or 0.1% DMSO (50 µL for all). After 5 min of auraptene treatment, a 0.7 mg/g dose of ADP was injected via the tail vein. Blood (0.5 mL) was collected by cardiac puncture and transferred into BD microtainer^®^ tubes with K_2_EDTA for the determination of platelet counts using an automatic cell counter (Procyte Dx; IDEXX Laboratories Inc., Westbrook, ME, USA). The rate of mortality was determined in each group.

### 4.10. Statistical Analysis

The data are presented as mean ± SEM, and convoyed by the number of interpretations (*n*). Specifically, *n* represents the number of investigates, and each investigation was accompanied using different blood donors. The unpaired Student’s *t*-test and analysis of variance (ANOVA) were used to determine the significance of differences among the groups. Groups with significant differences in analysis were compared using the Student–Newman–Keuls method. *p* < 0.05 indicated statistical significance.

## Figures and Tables

**Table 1:** Effect of aspirin and auraptene on mortality and platelet count of acute pulmonary thrombosis caused by intravenous injection of adenosine diphosphate (ADP) in experimental mice.

	Total Number	Number of Deaths	Mortality (%)	Platelet Count (K/μL)
DMSO	8	0	0	915 ± 24
ADP (0.7 mg/g)				
+ DMSO	8	7	87.5	770 ± 22 **
+ aspirin (mg/kg)				
20	8	2	25.0	888 ± 29 ^#^
+ auraptene (mg/kg)				
7.5	8	5	62.5	819 ± 21
15	8	2	25.0	879 ± 26 ^#^

Platelet count was presented as means ± SEM (*n* = 8). ** *p* < 0.01 compared with the DMSO group; ^#^ *p* < 0.05 compared with the DMSO + ADP group.
